# Case Fatality Rate and Length of Hospital Stay among Patients with Typhoid Intestinal Perforation in Developing Countries: A Systematic Literature Review

**DOI:** 10.1371/journal.pone.0093784

**Published:** 2014-04-17

**Authors:** Vittal Mogasale, Sachin N. Desai, Vijayalaxmi V. Mogasale, Jin Kyung Park, R. Leon Ochiai, Thomas F. Wierzba

**Affiliations:** 1 Policy and Economic Research Centre, Access Department, Development and Delivery Unit, International Vaccine Institute, Seoul, South Korea; 2 Clinical Development and Regulatory Department, Development and Delivery Unit, International Vaccine Institute, Seoul, South Korea; 3 Epidemiology Department, Development and Delivery Unit, International Vaccine Institute, Seoul, South Korea; 4 Biostatistics and Data Management Department, Development and Delivery Unit, International Vaccine Institute, Seoul, South Korea; 5 International Vaccine Institute, Seoul, South Korea; 6 Development and Delivery Unit, International Vaccine Institute, Seoul, South Korea; State Key Laboratory of Pathogen and Biosecurity, Beijing Institute of Microbiology and Epidemiology, China

## Abstract

**Background:**

Typhoid fever remains a major health problem in the developing world. Intestinal perforation is a lethal complication and continues to occur in impoverished areas despite advances in preventive and therapeutic strategies.

**Objectives:**

To estimate the case fatality rate (CFR) and length of hospital stay among patients with typhoid intestinal perforation in developing countries.

**Data Sources:**

Peer-reviewed publications listed in PubMed and Google Scholar.

**Study Eligibility:**

The publications containing data on CFR or length of hospitalization for typhoid fever from low, lower middle and upper middle income countries based on World Bank classification. Limits are English language, human research and publication date from 1st January 1991 to 31st December 2011.

**Participants:**

Subjects with reported typhoid intestinal perforation.

**Interventions:**

None, standard practice as reported in the publication.

**Study Appraisal and Synthesis Methods:**

Systematic literature review followed by meta-analysis after regional classification on primary data. Descriptive methods were applied on secondary data.

**Results:**

From 42 published reports, a total of 4,626 hospitalized typhoid intestinal perforation cases and 706 deaths were recorded (CFR = 15·4%; 95% CI; 13·0%–17·8%) with a significant regional differences. The overall mean length of hospitalization for intestinal perforation from 23 studies was 18.4 days (N = 2,542; 95% CI; 15.6–21.1).

**Limitations:**

Most typhoid intestinal perforation studies featured in this review were from a limited number of countries.

**Conclusions:**

The CFR estimated in this review is a substantial reduction from the 39.6% reported from a literature review for years 1960 to 1990. Aggressive resuscitation, appropriate antimicrobial coverage, and prompt surgical intervention may have contributed to decrease mortality.

**Implications:**

The quantification of intestinal perforation outcomes and its regional disparities as presented here is valuable in prioritizing and targeting typhoid-preventive interventions to the most affected areas.

## Introduction

Typhoid fever is caused by the gram negative bacillus *Salmonella enterica* serovar Typhi (*S. typhi*) [1] continues to be a public health problem in developing countries [2]. It is transmitted via the faeco-oral route through ingestion of contaminated food or water. The disease is characterized by prolonged fever, and constitutional symptoms including headache, anorexia and abdominal pain [3]. The systemic involvement in typhoid fever can result in extra-intestinal complications such as encephalopathy, meningitis, hepatitis, myocarditis and pneumonia, while the most common gastro-intestinal complication is haemorrhage [4]. Intestinal perforation is a potentially fatal complication of typhoid fever secondary to the inflammation and necrosis of Peyer's patches when not treated early and appropriately. Generally, perforation is a late complication occurring in the third week of illness, though it is reported earlier in second week in developing countries for reasons that are not completely understood [4], [5], [6], [7].

Case fatality rate (CFR) in intestinal perforation is dependent on various factors such as the quality of health care service received, characteristics of the organism and host factors. The diagnostic and therapeutic management for intestinal perforation have changed significantly over the past three decades which has potential implications on morbidity, mortality, hospital costs and societal costs. Aggressive resuscitation and prompt surgical intervention within the first 24 hours of perforation, along with appropriate antimicrobial coverage, are considered key measures in intestinal perforation management in recent days [8], [9], [10], [11]. The appearance and spread of multidrug resistant *S. typhi* strains is another factor that influences outcomes of typhoid intestinal perforation. Multidrug resistant strains exhibiting resistance to ampicillin, chloramphenicol, trimethoprim-sulfamethoxazole have emerged in South East Asia since the late 1980s. They have subsequently spread to other regions of the world, affecting morbidity, mortality and duration of treatment [12], [13], [14]. Poorer outcomes have been observed in those with late presentation (≥24 hours since perforation) [9], [15], multiple perforations (>1) [11], [15], [16], [17], and post- operative complications such as faecal fistula [15], [18].

A worldwide case series from 1960–90 had reported 1,990 cases of intestinal perforation in 66,157 patients with typhoid fever [19]. The publication reported a 3% perforation rate and a 39.6% CFR among typhoid perforation cases, noting an overall male preponderance. Advanced perforation management and emerging multidrug-resistant *S. typhi* in recent years could potentially modify characteristics of typhoid intestinal perforation and CFR. We present an updated review of CFR, age and gender characteristics, and length of hospital stay associated with typhoid intestinal perforation by geographical regions from articles published from 1991 to 2011 in low income, lower middle income and upper middle income countries [20].

## Methodology

### Search strategy and selection criteria

A systematic literature review was carried out using PubMed electronic database for typhoid fever intestinal perforation related publications in English from 1^st^ January 1991 to 31^st^ December 2011. The search was repeated using the Google Scholar electronic database for additional publications. The key terms used in the search were “Typhoid Fever”, “Enteric Fever”, “Salmonella Typhi”, and “Intestinal Perforation”. The selection criteria and search terms for the study inclusion are listed in Table 1. Two researchers conducted independent reviews based on defined search strategy and criteria, and compared the results before selecting final papers. One researcher extracted data and another researcher matched the data with original papers to verify for its correctness. No written protocol was developed.

**Table 1:** Selection criteria for study inclusion.

**Selection criteria**
• Published in English in peer reviewed journal from 1^st^ January 1991 to 31^st^ December 2011
• Presents typhoid intestinal perforation case fatality rate or length of stay in hospitalized patients
• Includes data at least from 1990 or afterwards
• Contains data from low income, lower middle income and upper middle income countries based on World Bank classification- “Country and Lending Groups by income”, 2011 [20].
**Search terms in PubMed**
• ((Typhoid fever) OR (Enteric Fever) OR (Salmonella Typhi)) AND ((Intestinal perforation) OR (perforation))

### Analytical method

An important aspect of this review was to capture the regional differences in CFR due to typhoid intestinal perforation, age and gender characteristics, and length of hospital stay. To identify the regional differences, we categorized the studies by geographical regions, namely: Asian countries, African countries and countries in other regions.

We have deployed meta-analysis approach [21] for validating and summarizing results so that a regional comparison of variables can be made. In this review, we have applied heterogeneity test to compare dissimilarity between results extracted from various primary studies based on a random effect model. The heterogeneity test was utilised to verify the validity of results and to potentially eliminate the effect of study quality and publication bias (the association of publication probability with the statistical significance of study results) [22], [23]. We considered the results of various studies comparable if no heterogeneity in the results was observed.

To perform meta-analysis, first, data on CFR, male to female ratio, age and length of stay in hospitalized intestinal perforation cases were extracted from selected publications. The data was classified by three geographical regions and listed in descending chronological order of year of publication. Then, the test for heterogeneity was conducted for CFR and male to female ratio to explore the true effects. The mean and corresponding 95% confidence interval for individual studies were estimated based on a random effect model and a graphical overview of the results was obtained by forest plot. We did not estimate 95% confidence interval of individual studies and heterogeneity test by using meta-analysis for age and length of hospital stay as variance was not available.

The validated results from meta-analysis for various regions were compared using Kruskal-Wallis test [24] to assess whether there was a significant difference in results from different geographical regions. If a significant difference between regions was observed, a simultaneous multiple paired comparison was performed to test inequalities between three possible combinations of two regions each. We applied nonparametric Kruskal-Wallis test on CFR, male to female ratio, age and length of hospital stay to compare the three regions. The box plot was generated to show data dispersion within each region and the strength of linear relationship between time and perforation outcome was tested using Spearman correlation coefficient [25], [26].

The analysis was performed using statistical software R while all statistical comparisons were tested for overall significance level at 5% (alpha = 0.05).

The selected papers had some additional information on the characteristics of intestinal perforation such as presenting symptoms, duration of illness, management procedures and post-operative complications. These features were summarized using descriptive methods.

## Results

**Figure 1:**
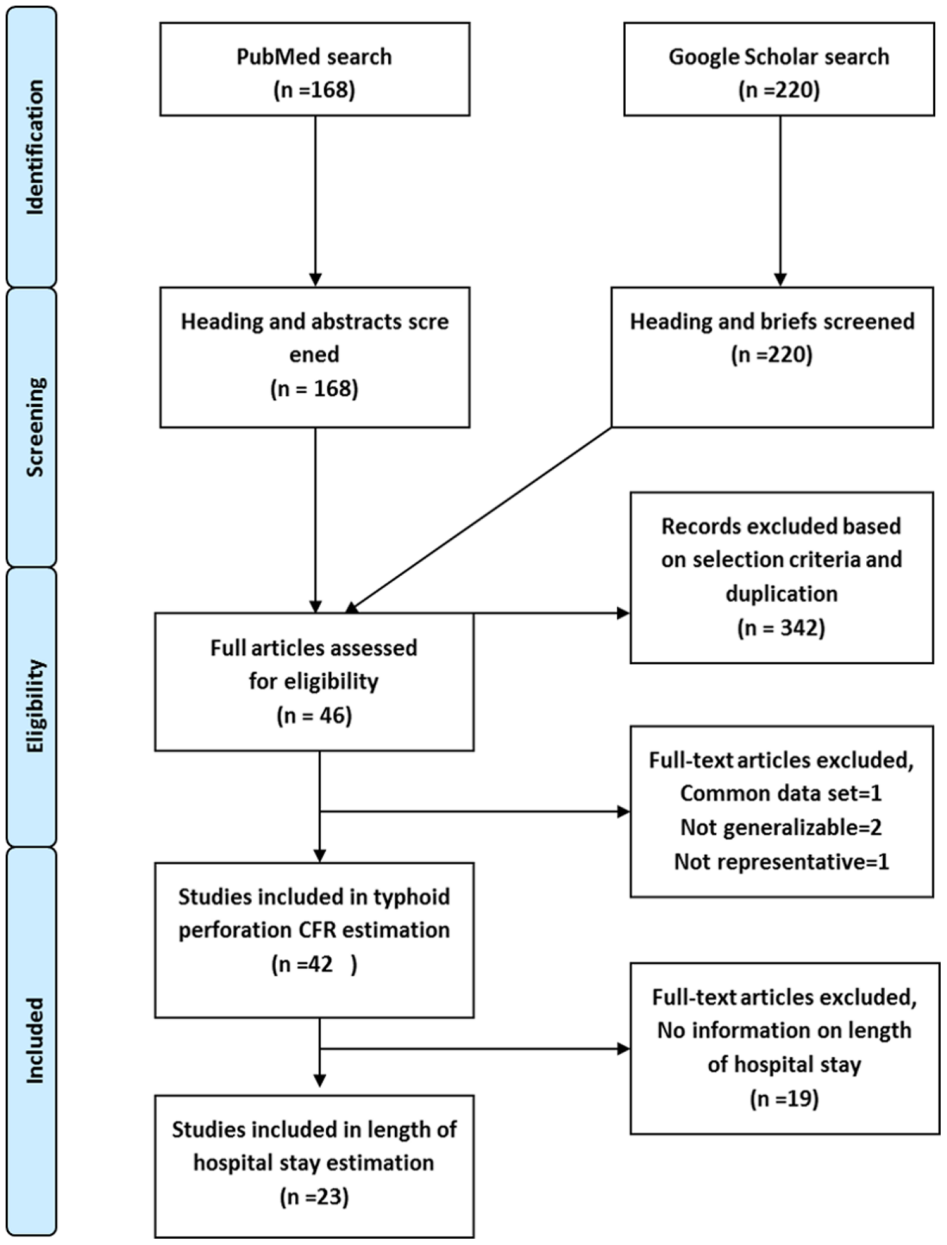
Study selection flow diagram. CFR = case fatality rate.

A total of 3,941 results on typhoid fever were narrowed down to 168 publications pertaining to typhoid intestinal perforation. When 168 abstracts were reviewed, we found 37 eligible papers based on the selection criteria. Supplementary literature search using Google Scholar identified 220 papers from which 9 additional papers were found eligible for the review (Figure 1).

Upon review of the 46 papers identified from low income, lower middle income and upper middle income countries, we found two of the papers analysed the same data set [27], [28]. We chose the article with the most comprehensive information [28]; noted in the figure 1 as exclusion based on common dataset. An additional two papers were excluded as they had presented CFR in specific surgical procedures that could not be generalized [29], [30]; noted in the figure 1 as exclusion based on being not generalizable. One study presenting 12 typhoid perforation cases in a tertiary hospital in Nigeria with an outlier CFR was excluded since the study population was not representative. These subjects were referred late, presenting in critically ill condition after treatment failure [31]. Of the remaining 42 papers included in final review (Table 2) [5], [6], [7], [11], [15], [16], [17], [18], [32], [33], [34], [35], [36], [37], [38], [39], [40], [41], [42], [43], [44], [45], [46], [47], [48], [49], [50], [51], [52], [53], [54], [55], [56], [57], [58], [59], [60], [61], [62], [63], twelve articles contained data from before and after 1990 [6], [7], [15], [35], [45], [46], [48], [49], [58], [60], [62], [63]. Though the analysis predominantly included data after 1990, the complete period ranged from 1978–2010. Of these 42 papers, 23 presented data on length of hospital stay (Table 3) [5], [6], [7], [11], [16], [17], [18], [28], [32], [33], [34], [35], [37], [39], [41], [42], [44], [45], [50], [52], [60], [61], [63].

**Table 2:** Case fatality rate (CFR) in hospitalized typhoid intestinal perforation cases by study (1991–2011).

Country	Year of publication	Study period	Hospitalized typhoid perforation cases	Deaths	CFR	Reference
**Africa**						
Nigeria	2011	2002–2009	153	16	10.5%	[18]
Nigeria	2010	2004–2008	46	13	28.3%	[32]
Ghana	2009	2000–2005	650	82	12.6%	[33]
Nigeria	2008	1990–2004	105	14	13.3%	[34]
Nigeria	2008	1995–2004	89	17	19.1%	[17]
Nigeria	2007	1996–2005	184	42	22.8%	[5]
Nigeria	2007	1980–2005	20	4	20.0%	[35]
Ghana	2007	2001–2005	522	61	11.7%	[36]
Nigeria	2007	2004–2006	53	8	15.1%	[11]
Ghana	2007	2002–2005	248	27	10.9%	[37]
Togo	2005	2003	112	18	16.1%	[38]
Nigeria	2005	1994–2003	101	14	13.9%	[39]
Nigeria	2004	1997–2003	47	6	12.8%	[40]
Ivory Coast	2004	1995–1998	64	22	34.4%	[41]
Nigeria	2004	1996–2000	43	6	14.0%	[42]
Nigeria	2003	1998–2001	105	17	16.2%	[43]
Nigeria	2003	1990–2000	108	27	25.0%	[44]
Nigeria	2002	1989–1998	60	15	25.0%	[45]
Nigeria	2001	1984–1999	106	25	23.6%	[6]
Ethiopia	2000	1984–1995	27	10	37.0%	[46]
Nigeria	1999	1987–1996	64	25	39.1%	[7]
Nigeria	1998	1991–1994	75	15	20.0%	[47]
Nigeria	1997	1989–1990	50	14	28.0%	[15]
Nigeria	1992	1986–1990	18	5	27.8%	[48]
Ghana	1992	1978–1991	195	61	31.3%	[49]
**Asia**						
Pakistan	2009	2003–2008	44	6	13.6%	[50]
Pakistan	2006	2003–2005	112	8	7.1%	[51]
Nepal	2006	2002–2004	102	7	6.9%	[16]
Pakistan	2006	1998–2000	32	4	12.5%	[52]
Pakistan	2005	2002–2004	72	10	13.9%	[53]
Vietnam	2004	1997–1998	27	1	3.7%	[54]
India	2003	2000–2001	200	21	10.5%	[55]
India	2001	1990–1998	100	7	7.0%	[9]
Pakistan	2000	1991–1994	76	17	22.4%	[56]
India	1997	1990–1995	110	18	16.4%	[28]
Pakistan	1997	1994–1995	140	12	8.6%	[57]
India	1994	1987–1990	65	13	20.0%	[58]
**Others**						
Turkey	2010	1994–2010	22	1	4.6%	[59]
Turkey	2007	1978–2004	82	9	11.0%	[60]
Turkey	2002	1990–2000	42	2	4.8%	[61]
Mexico	1998	1985–1994	116	2	1.7%	[62]
Turkey	1995	1987–1993	39	4	10.3%	[63]
**Overall**			**4,626**	**706**		

Note: Studies are listed in the table by descending order of publication year.

**Table 3:** Length of hospital stay among typhoid intestinal perforation cases by study (1991–2011).

Country	Age range (mean)	Number of cases	Mean length of stay (days)	Range of length of stay	Study period	Year of publication	Reference
**Africa**							
Nigeria	3–15 (9.6)	153	21.0	8–67	2002–2009	2011	[18]
Nigeria	9–15 (9.5)	46	22.9	4–46	2004–2008	2010	[32]
Ghana	1–15 (8.8)	650	14.4	1–77	2001–2005	2009	[33]
Nigeria	15–72 (27)	105	16.1	NA	1990–2004	2008	[34]
Nigeria	1–15 (9.1)	89	22.0	18–42	1995–2004	2008	[17]
Nigeria	4–15 (6.8)	184	35.8	8–196	1996–2005	2007	[5]
Nigeria	2–55 (12.3)	53	16.1	8–57	2004–2006	2007	[11]
Nigeria	11–45 (25.7)	20	16.3	0–32	1980–2005	2007	[35]
Ghana	16–55 (24.9)	248	12.4	5–46	2002–2005	2007	[37]
Nigeria	4–85 (19.9)	101	18.0	NA	1994–2003	2005	[39]
Ivory Coast	5–63 (34)	64	30.0	8–52	1995–1998	2004	[41]
Nigeria	6–32 (15.9)	43	21.6	8–74	1996–2000	2004	[42]
Nigeria	3–14 (8.8)	105	24.6	11–54	1990–2000	2003	[44]
Nigeria	1–67 (24.3)	60	11.7	1–60	1989–1998	2002	[45]
Nigeria	3–14 (10)	106	23.6	NA	1984–1999	2001	[6]
Nigeria	1–12 (8)	64	20.6	12–48	1989–1998	1999	[7]
**Asia**							
Pakistan	10–45 (29.4)	44	17.6	NA	2003–2008	2009	[50]
Nepal	14–78 (28.4)	102	8.5	NA	2002–2004	2006	[16]
Pakistan	15–30	32	16.5	NA	1998–2000	2006	[52]
India	5–58 (24.0)	110	15.8	NA	1990–1995	1997	[28]
**Others**							
Turkey	7–68 (36.3)	82	11.7	5–25	1978–2004	2007	[60]
Turkey	4–14 (10.4)	42	13.4	7–23	1990–2000	2002	[61]
Turkey	3–76	39	12.0	NA	1987–1993	1995	[63]

SD = standard deviation, NA = not available. Note: Studies are listed in the table by descending order of publication year.

The majority of publications came from five countries: Nigeria, Ghana, Pakistan, India and Turkey. We categorized 4,626 typhoid intestinal perforation cases from 11 countries into three geographical regions, Africa, Asia and others (Table 2). Two studies that assessed socio-economic status reported that around 78% to 88% of typhoid intestinal perforation cases were observed among people from low socio-economic strata [39], [45].

The common presenting symptoms of typhoid intestinal perforation in the reviewed papers were fever, abdominal pain, diarrhoea, constipation, vomiting and abdominal distension. The mean duration from the onset of illness to the presentation of typhoid perforation at hospital was 10.6 days (SD = 2.1, min = 7.4, max = 15.7) from 1,925 cases reported in 25 studies. Over the years, aggressive surgical procedures became popular over conservative methods and simple drainage [8], [9], [27], [48], [63]. Among 26 papers that described surgical procedures, 23 reported simple closure or two layered closure as the most practiced surgical procedure. Solitary perforations were observed in 76% of the operated typhoid perforation cases that reported number of perforations per case (N = 2,903), while the remaining cases involved multiple perforations. Postoperative complications were common and were reported in at least 57% of the 2,063 cases that described the complications. The common complications were: wound infection, wound dehiscence (breaking open of the wound along surgical suture), persistent peritonitis, intra-abdominal abscess and entero-cutaneous fistula, all of which were often associated with increased hospital stay [6]. Around 18% (140/792) of operated perforation cases were re-operated due to the complications [17], [18], [32], [34], [37], [47], [56] while, re-perforation was observed during the surgery in half (52/98) of the reported re-operated cases [37], [56].

**Figure 2:**
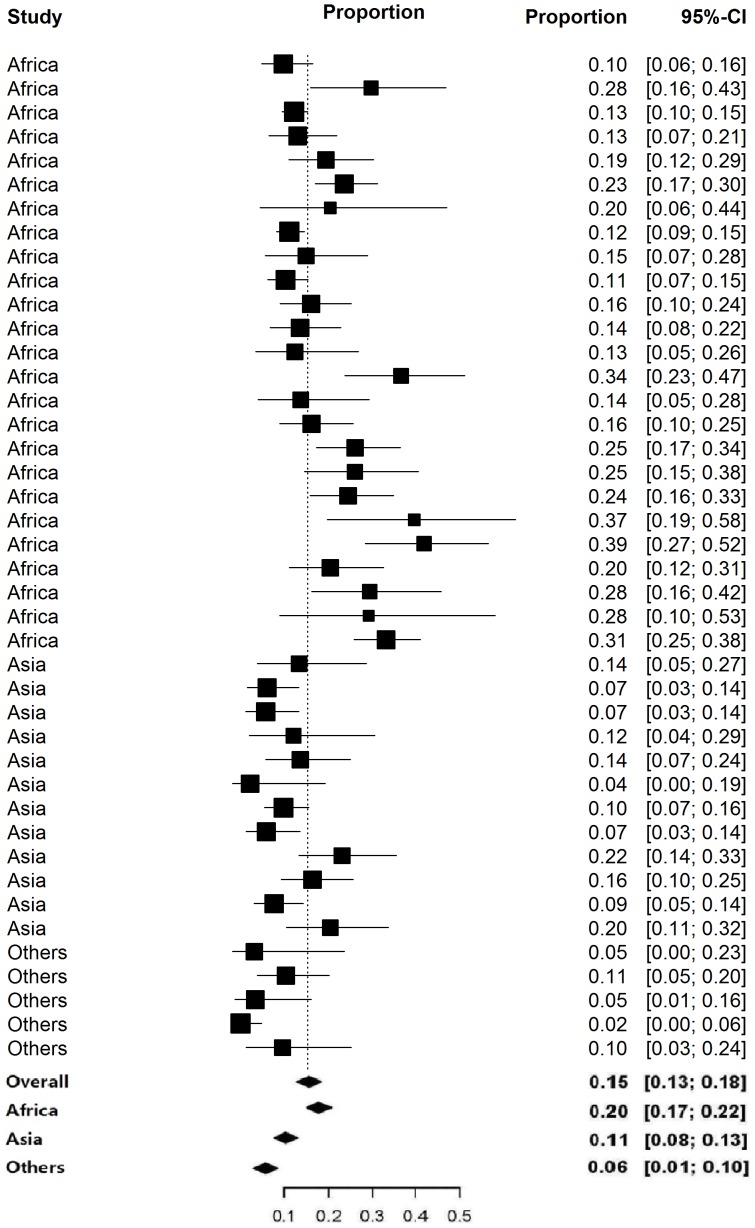
Forest plot showing mean case fatality rate for intestinal perforation along with 95% confidence interval based on studies published from 1991 to 2011.

**Figure 3:**
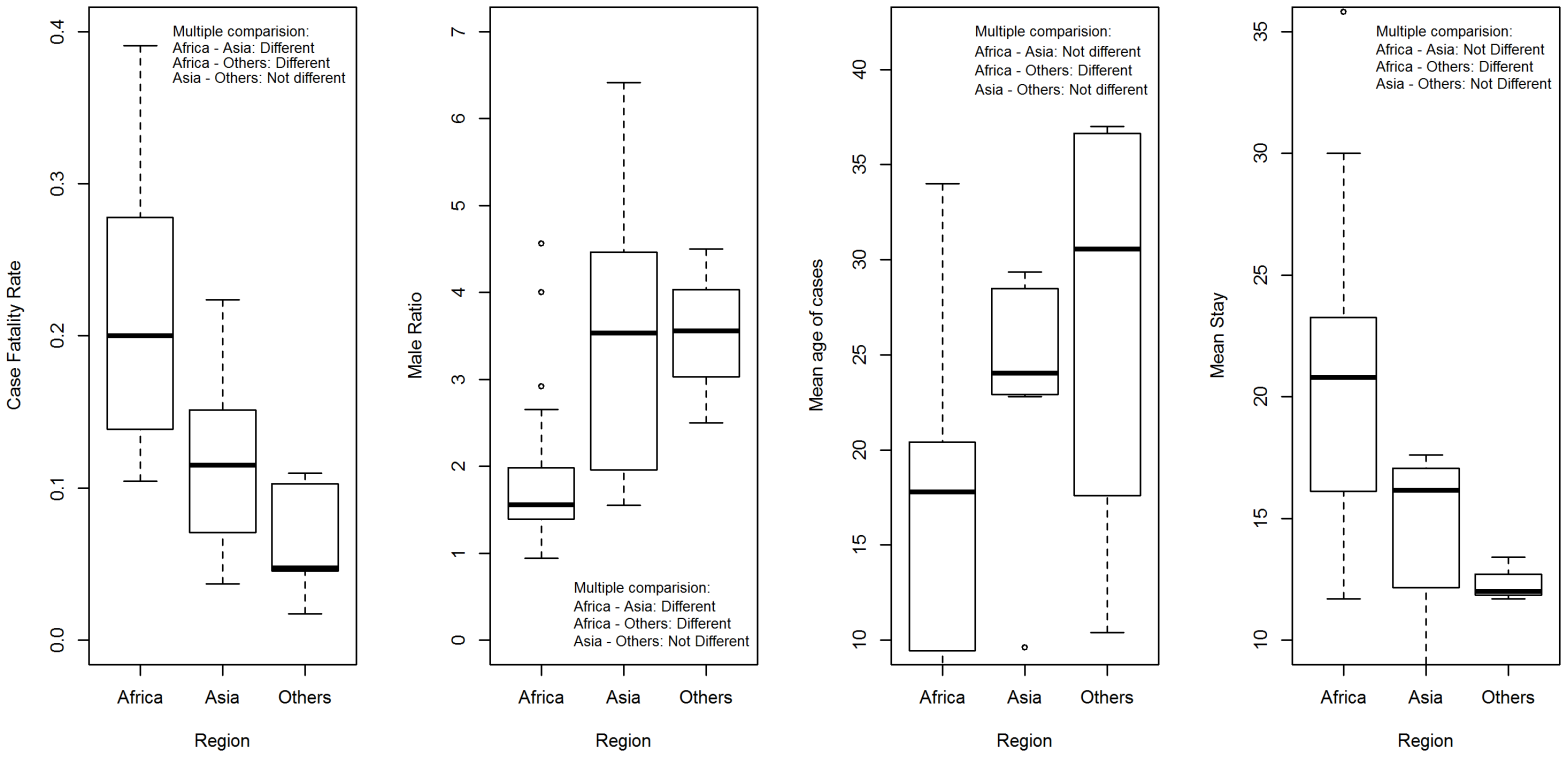
Box plot showing multiple comparisons between regions for case fatality rate, male to female ratio, mean age and mean length of hospital stay based on studies published from 1991 to 2011.

The overall mean CFR among typhoid perforation cases was 15.4% (95% CI; 13.0%–17.8%), with the highest rates observed in African countries (19.5%; 95% CI; 16.6%–22.4%) followed by Asian countries (10.7%; 95% CI; 8.0%–13.4%) and countries from other regions (5.55%; 95% CI; 1.45%–9.65%), (Table 4). The meta-analysis showed a significant heterogeneity in between the published studies in both Africa and Asia (Table 4) and the forest plot (Figure 2) indicated that point estimates do not have high credence. Overall mean CFR between three regions was significantly different as shown by nonparametric Kruskal-Wallis test (Table 4). The box plot showed that the CFR of Africa derived the significant difference from that of other two regions (Figure 3).

**Table 4:** Mean values for weighted case fatality rate, male to female ratio, age and length of hospital stay by region in studies published from 1991 to 2011.

Variables	Region	No. of Studies	Total (N)	Average (N)	Mean [95% CI]	Heterogeneity (p-value)	p-value§
Case fatality rate*	Overall	42	4626	110.1	0.154 [0.130; 0.178]	<0.001	<0.001
	Africa	25	3245	129.8	0.195 [0.165; 0.224]	<0.001	
	Asia	12	1080	90.0	0.107 [0.080; 0.134]	0.016	
	Others	5	301	60.2	0.055 [0.014; 0.096]	0.062	
Male to female ratio*	Overall	40	4456	111.4	2.160 [1.151; 4.031]	1.0	0.001
	Africa	25	3230	129.2	1.716 [0.777; 3.786]	1.0	
	Asia	12	1080	90.0	3.094 [0.986; 9.705]	1.0	
	Others	3	146	48.7	3.406 [0.339; 34.271]	0.979	
Weighted age (year)	Overall	35	4016	114.7	19.286 [16.329; 22.244]		0.023
	Africa	24	3218	134.1	16.697 [13.605; 19.787]		
	Asia	7	536	76.6	23.684 [17.385; 29.983]	NA	
	Others	4	262	65.5	27.130 [7.283; 46.977]		
Mean stay (day)	Overall	23	2542	110.5	18.374 [15.624; 21.124]		0.030
	Africa	16	2091	130.9	20.443 [17.065; 23.821]		
	Asia	4	288	72.0	14.600 [8.022; 21.177]	NA	
	Others	3	163	54.3	12.367 [10.113; 14.621]		

N = sample size, NA = not applicable,

* weighted mean,

§ region comparison by Kruskal-Wallis test.

**Figure 4:**
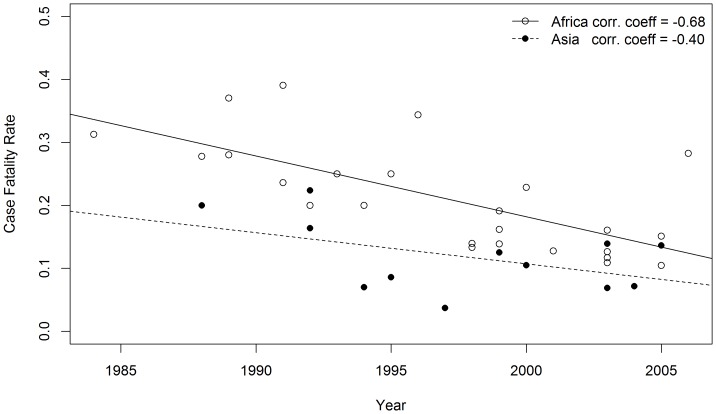
Trends of typhoid intestinal perforation case fatality rate in Africa and Asia from studies published from 1991 to 2011. Spearman Correlation coefficient was used to estimate strength of association.

To estimate trends in CFR, a scatter plot of CFR in patients with perforation against time was drawn for African and Asian regions applying the rate to the final year of the study period. The Spearman correlation coefficient of CFR were −0.68 (*p*<0.01) and −0.4 (*p* = 0.2) for Africa and Asia respectively which indicates a declining trend in CFR of typhoid intestinal perforation (Figure 4). The decline is statistically significant in African region only.

Papers presenting information on gender (n = 40), suggested that males were 2.16 times more likely to be hospitalized compared to females (95% CI = 1.1–4.0) for typhoid intestinal perforation (Table 4). The male to female ratio was significantly higher in Asia and other regions compared to Africa, while there was no statistical difference between male to female ratio of hospitalized typhoid intestinal perforation cases between Asia and other regions (Figure 3).

Based on 35 studies reporting age data, the unweighted mean age of hospitalization among all patients was 19.3 years. The overall difference in reported age at hospitalization for intestinal perforation is significantly different between the regions (*p* = 0.023, Table 4). African region displayed a significantly lower mean age at hospitalization (Figure 3) compared to other regions.

Based on the review of 23 relevant papers, the longest duration of hospitalization following intestinal perforation was estimated in African region (20.4 days). The overall unweighted mean length of hospitalization from all studies was 18.4 days (Table 4). The mean of length of hospital stay was significantly higher in Africa compared to average length of hospital stay in other regions (Figure 3).

Several investigators in the African region have followed up intestinal perforation cases for a longer duration. They have reported deaths due to malnutrition, resulting either from an entero-cutaneous fistula or peristomal ulcerations [6], [34], [41]. Development of entero-cutaneous fistula was strongly linked with increased mortality [15], [18], [28], [34]. While most studies describe deaths occurring within a few weeks following intestinal perforation, a cohort of 64 postoperative cases found considerable mortality of 3–6 months (64%) and after 6 months (27%) secondary to postoperative complications [41].

## Discussion

The review suggests a substantial reduction in typhoid intestinal perforation CFR from 39.6% reported from a literature review conducted for years 1960 to 1990 to 15.4% for years 1991 to 2011. The declining CFR trends in Africa and Asia within current review period indicates a gradual fall over the time. Based on a three-decade study, Chatterjee et al noted a declining trend of CFR in hospitalized typhoid perforation cases in India from 47.2% (1966–78) to 17.7% (1981–88) and 7% (1990–98) [9]. Our review denotes that the falling trend of typhoid intestinal perforation CFR is not confined to India, but also evident in other developing countries. As illustrated in the Indian case review mentioned above, improved typhoid perforation management practices over the years may be responsible for the positive effect in reducing the CFR and possibly offsetting increased complications due to multidrug resistance. Improving access to diagnosis, treatment and case management could further contribute to decrease in CFR.

While declining over the last five decades, this review indicates that even today more than one in 10 patients with typhoid intestinal perforation in Africa and Asia will die. The review also implies a high variability of perforation CFR within each of Africa, Asia and other regions; yet African region has significantly higher CFR. Not only people hospitalized for typhoid intestinal perforation seems younger in Africa compared to Asia and other regions, but also appear to stay longer in hospitals for treatment. The review suggests typhoid intestinal perforation occurs most commonly in the second week of illness, and is associated with a high proportion of postoperative complications.

This review has four important global public health implications. First, as noted above, typhoid intestinal perforation CFR is high even today in Africa and Asia, alerting to the importance of typhoid prevention and control activities in those regions. Second, it suggests that there is an opportunity to reduce typhoid intestinal perforation burden by improving access to care and management, such as aggressive resuscitation, better surgical facilities and use of appropriate antibiotics. Third, it implies that a higher morbidity is likely to be shouldered in countries with increased length of hospital stays where younger people are affected. This is based on the fact that these longer hospital stays are associated with more severe disease or complications [6], [11], [34], [37], which also imply higher treatment costs, and greater loss of productivity. Thus morbidity of typhoid could be significant enough to warrant prevention activities in some regions where even if incidence is not that high. Fourth, it provides some basic information needed for estimating disease and economic burden of typhoid in developing countries. The CFR is useful in model based estimation of typhoid intestinal perforation deaths at the country and regional levels. Length of hospital stay is useful in estimating direct medical costs and productivity loss associated with typhoid intestinal perforation. The significant difference in the characteristics of typhoid intestinal perforation between the regions, particularly between Africa and Asia, necessitates the need for separate regional data inputs in modelling the disease and economic burden.

This review also brings out an important research agenda. We noted delayed deaths even beyond six months occurring due to intestinal perforation, particularly as a result of malnutrition. However, most studies did not report follow-up results of patients for a long enough time to properly identify the delayed deaths. It is important to follow-up typhoid intestinal perforation cases for sufficient time to better understand the CFR in future studies.

Most intestinal perforation studies featured in this review are from one of three countries per continent. Nigeria, Pakistan and Turkey over represent Africa, Asia and the other regions, respectively. Thus, the regional average could be influenced by these selected countries in their respective sub-regions: West Africa, the Indian subcontinent and the Middle East. There were only a few studies from other regions which may bias the ability to draw meaningful conclusions due to limited data. The analysis includes only English literature and hence information published in other languages is not represented. This exclusion bias affects data from francophone Africa, from where only a handful of studies were published in English. Twenty three studies published in languages other than English were excluded before screening, some of which might have contained data on typhoid intestinal perforation.

Because some studies containing data before and after the 1990 cut-off time point were included in the analysis, it should be noted that some data before the cut-off time (<1990) could have been included in the review. Though these reports did not separate the data by decade, the bulk of the data was within the review period (>1990). For this reason, we feel that the majority of cases reflect the time period of interest.

We may have slightly underestimated the length of hospital stay for intestinal perforation cases. Length of hospital stay could be shorter for those who died due to intestinal perforation than those who survived. Some of the studies presented here do not classify length of stay by survival state and therefore reports are inclusive of those who have died which are likely to be an underestimate.

## Conclusion

This review estimated an overall average case fatality rate of 15.4% among hospitalized intestinal perforation cases with higher case fatality rates in African region followed by Asia. The results imply that younger people are hospitalized for typhoid intestinal perforation in Africa and remain hospitalized longer compared to Asia and other developing regions. Typhoid prevention activities such as the provision of clean water, sanitation, personal hygiene measures, and vaccination should be prioritized in Africa as well as in Asia to limit number of deaths resulting from typhoid intestinal perforation. We emphasize the need for a regional approach in typhoid research, prevention and control activities. Estimation of regional typhoid disease and economic burden would be valuable in informing resource allocation strategies aimed at preventing and controlling typhoid.

## Supporting Information

Checklist S1 **PRISMA checklist.**
(DOC)

## References

[1] Bhutta ZA (2006) Current concepts in the diagnosis and treatment of typhoid fever. BMJ 333: 78–82. 16825230 10.1136/bmj.333.7558.78 PMC1489205

[2] Crump JA, Luby SP, Mintz ED (2004) The global burden of typhoid fever. Bull World Health Organ 82: 346–353. 15298225 PMC2622843

[3] Parry CM, Hien TT, Dougan G, White NJ, Farrar JJ (2002) Typhoid fever. N Engl J Med 347: 1770–1782. 12456854 10.1056/NEJMra020201

[4] WHO (2003) Background document: The diagnosis, treatment and prevention of typhoid fever. World Health Organization. Geneva, Switzerland.

[5] Uba AF, Chirdan LB, Ituen AM, Mohammed AM (2007) Typhoid intestinal perforation in children: a continuing scourge in a developing country. Pediatr Surg Int 23: 33–39. 17086425 10.1007/s00383-006-1796-3

[6] Rahman GA, Abubakar AM, Johnson AW, Adeniran JO (2001) Typhoid ileal perforation in Nigerian children: an analysis of 106 operative cases. Pediatr Surg Int 17: 628–630. 11727054 10.1007/s003830100008

[7] Ameh EA (1999) Typhoid ileal perforation in children: a scourge in developing countries. Ann Trop Paediatr 19: 267–272. 10715713 10.1080/02724939992356

[8] Ukwenya AY, Ahmed A, Garba ES (2011) Progress in management of typhoid perforation. Ann Afr Med 10: 259–265. 22064250 10.4103/1596-3519.87040

[9] Chatterjee H, Jagdish S, Pai D, Satish N, Jayadev D, et al (2001) Changing trends in outcome of typhoid ileal perforations over three decades in Pondicherry. Trop Gastroenterol 22: 155–158. 11681112

[10] Richens J (1991) Management of bowel perforation in typhoid fever. Trop Doct 21: 149–152. 1746032 10.1177/004947559102100405

[11] Edino ST, Yakubu AA, Mohammed AZ, Abubakar IS (2007) Prognostic factors in typhoid ileal perforation: a prospective study of 53 cases. J Natl Med Assoc 99: 1042–1045. 17913115 PMC2575870

[12] Bhutta ZA, Naqvi SH, Razzaq RA, Farooqui BJ (1991) Multidrug-resistant typhoid in children: presentation and clinical features. Rev Infect Dis 13: 832–836. 1962094 10.1093/clinids/13.5.832

[13] Mirza SH, Beeching NJ, Hart CA (1996) Multi-drug resistant typhoid: a global problem. J Med Microbiol 44: 317–319. 8636944 10.1099/00222615-44-5-317

[14] Bhutta ZA (1996) Impact of age and drug resistance on mortality in typhoid fever. Arch Dis Child 75: 214–217. 8976660 10.1136/adc.75.3.214 PMC1511710

[15] Adesunkanmi AR, Ajao OG (1997) The prognostic factors in typhoid ileal perforation: a prospective study of 50 patients. J R Coll Surg Edinb 42: 395–399. 9448395

[16] Karmacharya B, Sharma VK (2006) Results of typhoid perforation management: our experience in Bir Hospital, Nepal. Kathmandu Univ Med J (KUMJ) 4: 22–24. 18603862

[17] Ekenze SO, Okoro PE, Amah CC, Ezike HA, Ikefuna AN (2008) Typhoid ileal perforation: analysis of morbidity and mortality in 89 children. Niger J Clin Pract 11: 58–62. 18689141

[18] Nasir AA, Abdur-Rahman LO, Adeniran JO (2011) Predictor of mortality in children with typhoid intestinal perforation in a Tertiary Hospital in Nigeria. Pediatr Surg Int 10.1007/s00383-011-2924-2 21594718

[19] van Basten JP, Stockenbrugger R (1994) Typhoid perforation. A review of the literature since 1960. Trop Geogr Med 46: 336–339. 7892698

[20] WorldBank (2011) Country and Lending Groups: by income. World Bank

[21] Glass GV (1976) Primary, Secondary, and Meta-Analysis of Research. Educational Researcher 5: 3–8.

[22] Sutton AJ, Abrans KR, Jones DR, Sheldon TA (2000) Methods for Meta-analysis in Medical Research: John Wiley & Sons.

[23] Sterne HAC, Harbord RM (2004) Funnel plots in meta-analysis. The Stata Journal 4: 127–141.

[24] Kruskal WH, Wallis WA (1952) Use of Ranks in One-Criterion Variance Analysis. Journal of the American Statistical Association 47: 583–621.

[25] Spearman C (1904) The proof and measurement of association between two things. Amer J Psychol 15: 72–101. 3322052

[26] Hulley SB, Cummings SR, Browner WS, Grady DG, Newman TB (2007) Designing Clinical Research. Philadelphia: Wolters Kluwer Health/Lippincott Williams & Wilkins.

[27] Talwar S, Laddha BL, Jain S, Prasad P (1997) Choice of incision in surgical management of small bowel perforations in enteric fever. Trop Gastroenterol 18: 78–79. 9323924

[28] Talwar S, Sharma RK, Mittal DK, Prasad P (1997) Typhoid enteric perforation. Aust N Z J Surg 67: 351–353. 9193272 10.1111/j.1445-2197.1997.tb01990.x

[29] Singh KP, Singh K, Kohli JS (1991) Choice of surgical procedure in typhoid perforation: experience in 42 cases. J Indian Med Assoc 89: 255–256. 1795109

[30] Shukla VK, Sahoo SP, Chauhan VS, Pandey M, Gautam A (2004) Enteric perforation–single-layer closure. Dig Dis Sci 49: 161–164. 14992453 10.1023/b:ddas.0000011620.56077.97

[31] Osifo OD, Ogiemwonyi SO (2010) Typhoid ileal perforation in children in Benin city. Afr J Paediatr Surg 7: 96–100. 20431219 10.4103/0189-6725.62857

[32] Nuhu A, Dahwa S, Hamza A (2010) Operative management of typhoid ileal perforation in children. Afr J Paediatr Surg 7: 9–13. 20098001 10.4103/0189-6725.59351

[33] Abantanga FA, Nimako B, Amoah M (2009) The range of abdominal surgical emergencies in children older than 1 year at the Komfo Anokye Teaching Hospital, Kumasi, Ghana. Ann Afr Med 8: 236–242. 20139546 10.4103/1596-3519.59578

[34] Tade AO, Ayoade BA, Olawoye AA (2008) Pattern of presentation and management of typhoid intestinal perforation in Sagamu, South-West Nigeria: a 15 year study. Niger J Med 17: 387–390. 19048751 10.4314/njm.v17i4.37417

[35] Osime OC, Osifo OD (2007) Pattern and outcome of typhoid peforation in Benin city. Journal of Medicine and Biomedical Research 6: 13–18.

[36] Clegg-Lamptey JN, Hodasi WM, Dakubo JC (2007) Typhoid ileal perforation in Ghana: a five-year retrospective study. Trop Doct 37: 231–233. 17988489 10.1258/004947507782332784

[37] Oheneh-Yeboah M (2007) Postoperative complications after surgery for typhoid ileal perforation in adults in Kumasi. West Afr J Med 26: 32–36. 17595989 10.4314/wajm.v26i1.28300

[38] Saxe JM, Cropsey R (2005) Is operative management effective in treatment of perforated typhoid? Am J Surg 189: 342–344. 15792765 10.1016/j.amjsurg.2004.11.032

[39] Ugwu BT, Yiltok SJ, Kidmas AT, Opaluwa AS (2005) Typhoid intestinal perforation in north central Nigeria. West African journal of medicine 24: 1–6. 15909700 10.4314/wajm.v24i1.28152

[40] Edino ST, Mohammed AZ, Uba AF, Sheshe AA, Anumah M, et al (2004) Typhoid enteric perforation in north western Nigeria. Nigerian journal of medicine : journal of the National Association of Resident Doctors of Nigeria 13: 345–349. 15523859

[41] Kouame J, Kouadio L, Turquin HT (2004) Typhoid ileal perforation: surgical experience of 64 cases. Acta Chir Belg 104: 445–447. 15469159

[42] Naaya H, Eni U, Chama C (2004) Typhoid perforation in Maiduguri, Nigeria. Annals of African Medicine 3: 69–72.

[43] Agbakwuru EA, Adesunkanmi AR, Fadiora SO, Olayinka OS, Aderonmu AO, et al (2003) A review of typhoid perforation in a rural African hospital. West Afr J Med 22: 22–25. 12769301 10.4314/wajm.v22i1.27973

[44] Irabor DO (2003) Fifteen years of typhoid perforation in children in Ibadan: still a millstone around the surgeon's neck. The Nigerian J Sur Res 5: 92–99.

[45] Otegbayo JA, Daramola OO, Onyegbutulem HC, Balogun WF, Oguntoye OO (2002) Retrospective analysis of typhoid fever in a tropical tertiary health facility. Trop Gastroenterol 23: 9–12. 12170927

[46] Worku B (2000) Typhoid fever in an Ethiopian children's hospital: 1984–1995. Ethiop J Health Dev 14: 311–315.

[47] Meier DE, Tarpley JL (1998) Typhoid intestinal perforations in Nigerian children. World J Surg 22: 319–323. 9494426 10.1007/s002689900388

[48] Ndububa DA, Erhabor GE, Akinola DO (1992) Typhoid and paratyphoid fever: a retrospective study. Trop Gastroenterol 13: 56–63. 1413100

[49] Mock CN, Amaral J, Visser LE (1992) Improvement in survival from typhoid ileal perforation. Results of 221 operative cases. Ann Surg 215: 244–249. 1543396 10.1097/00000658-199203000-00008 PMC1242427

[50] Ansari AG, Naqvi SQH, Ghumro AA, Jamali AH, Talpur AA (2009) Management of typhoid ileal perforation: a surgical experience of 44 cases. Gom J Med Sc 7: 27–29.

[51] Malik AM, Laghari AA, Mallah Q, Qureshi GA, Talpur AH, et al (2006) Different surgical options and illeostomy in typhoid perforation. World J Med Sci 1: 112–116.

[52] Ahmed HN, Niaz MP, Amin MA, Khan MH, Parhar AB (2006) Typhoid perforation still a common problem: situation in Pakistan in comparison to other countries of low human development. J Pak Med Assoc 56: 230–232. 16767951

[53] Aziz M, Qadir A (2005) Faizullah (2005) Prognostic factors in typhoid perforation. J Coll Physicians Surg Pak 15: 704–707. 16300707

[54] Nguyen QC, Everest P, Tran TK, House D, Murch S, et al (2004) A clinical, microbiological, and pathological study of intestinal perforation associated with typhoid fever. Clin Infect Dis 39: 61–67. 15206054 10.1086/421555

[55] Beniwal US, Jindal D, Sharma J, Jain S, Shyam G (2003) Comparative study of operative procdures in typhoid perforation. Indian J Surg 65: 172–177.

[56] Khan A, Khan CH (2000) Typhoid enteric perforation. J Surgery Pakistan 5 (2) 37–39.

[57] Khan TM, Khan FM, Khan M, Shahabuddin, Khan OA (1997) Experience with typhoid ileal perforation. JPMI 11: 175–181.

[58] Gupta V, Gupta SK, Shukla VK, Gupta S (1994) Perforated typhoid enteritis in children. Postgrad Med J 70: 19–22. 8140012 10.1136/pgmj.70.819.19 PMC2397559

[59] Sumer A, Kemik O, Dulger AC, Olmez A, Hasirci I, et al (2010) Outcome of surgical treatment of intestinal perforation in typhoid fever. World J Gastroenterol 16: 4164–4168. 20806433 10.3748/wjg.v16.i33.4164 PMC2932920

[60] Atamanalp SS, Aydinli B, Ozturk G, Oren D, Basoglu M, et al (2007) Typhoid intestinal perforations: twenty-six year experience. World J Surg 31: 1883–1888. 17629741 10.1007/s00268-007-9141-0

[61] Onen A, Dokucu AI, Cigdem MK, Ozturk H, Otcu S, et al (2002) Factors effecting morbidity in typhoid intestinal perforation in children. Pediatr Surg Int 18: 696–700. 12598967 10.1007/s00383-002-0794-3

[62] Athie CG, Guizar CB, Alcantara AV, Alcaraz GH, Montalvo EJ (1998) Twenty-five years of experience in the surgical treatment of perforation of the ileum caused by Salmonella typhi at the General Hospital of Mexico City, Mexico. Surgery 123: 632–636. 9626313 10.1016/s0039-6060(98)70201-6

[63] Akgun Y, Bac B, Boylu S, Aban N, Tacyildiz I (1995) Typhoid enteric perforation. Br J Surg 82: 1512–1515. 8535806 10.1002/bjs.1800821120

